# Pushing the limits of accessible length scales via a modified Porod analysis in small-angle neutron scattering on ordered systems

**DOI:** 10.1107/S1600576724007295

**Published:** 2024-08-27

**Authors:** Xaver Simon Brems, Sebastian Mühlbauer, Robert Cubitt

**Affiliations:** a Large Scale Structures Group, Institut Laue–Langevin, 71 Avenue des Martyrs CS 20156, 38042Grenoble Cedex 9, France; b Heinz Maier-Leibnitz Zentrum (MLZ), Technische Universität München, Lichtenbergstrasse 1, 85748Garching, Germany; Universität Duisburg-Essen, Germany

**Keywords:** small-angle neutron scattering, Porod scattering, superconductivity, vortex matter

## Abstract

Small-angle neutron scattering is a widely used technique to study large-scale structures in bulk samples. The largest accessible length scale in conventional Bragg scattering is determined by the combination of the longest available neutron wavelength and smallest resolvable scattering angle. A method is presented that circumvents this limitation and is able to extract larger length scales from the low-*q* power-law scattering using a modification of the well known Porod law connecting the scattered intensity of randomly distributed objects to their specific surface area. It is shown that in the special case of a highly aligned domain structure the specific surface area extracted from the modified Porod law can be used to determine specific length scales of the domain structure. The analysis method is applied to study the micrometre-sized domain structure found in the intermediate mixed state of the superconductor niobium. The analysis approach allows the range of accessible length scales to be extended from 1 µm to up to 40 µm using a conventional small-angle neutron scattering setup.

## Abstract 2

This work presents a novel method for the analysis of small-angle scattering data from a highly aligned domain structure based on a modification of the well known Porod law. The analysis approach allows the range of accessible length scales to be extended from 1 µm to up to 40 µm using a conventional small-angle neutron scattering setup.

## 1. Introduction

Small-angle neutron scattering (SANS) is a widely used technique to study bulk samples with length scales in the range of 10–1000 nm. The isotope specificity of neutrons and their ability to interact with magnetic spins make SANS a powerful tool to determine the size and shape of structures in fields ranging from biology (Jeffries *et al.*, 2021 ▸) to magnetism and superconductivity (Mühlbauer *et al.*, 2019 ▸). However, the accessible length scales in a conventional SANS setup are limited by the available neutron wavelengths and resolvable scattering angles, with a rough upper limit for resolvable structures of approximately 1 µm. We present a novel method of circumventing this limitation using a modification of the well known Porod law in the case of aligned structures. We showcase our approach making use of the highly aligned domain morphology of the intermediate mixed state in the model superconductor niobium.

Superconductors are most commonly classified according to their response to an applied magnetic field. Type-I superconductors completely expel the magnetic field, called the Meissner state, while type-II superconductors allow for an additional state where the field can enter in a quantized manner in the form of flux lines, known as the mixed state. The flux lines are extended along the magnetic field direction and their mutual interaction is conventionally repulsive. Frequently, this leads to the formation of a hexagonal lattice consisting of flux lines that are aligned nearly parallel to the direction of the magnetic field. This structure, most commonly known as a flux line lattice (FLL), can be described by only two lattice vectors (Huebener, 2001 ▸). It is similar to other aligned periodic arrangements such as liquid crystals (Lee *et al.*, 2006 ▸).

A single flux line contains a quantum of magnetic flux. Therefore, the area density of flux lines is directly proportional to the mean internal field. This makes the FLL in superconductors an interesting model system, where we can tune the lattice spacing by changing the magnetic field. For example, at an applied field of 100 mT the inter flux line distance for a hexagonal lattice is approximately 150 nm.

**Figure 1:**
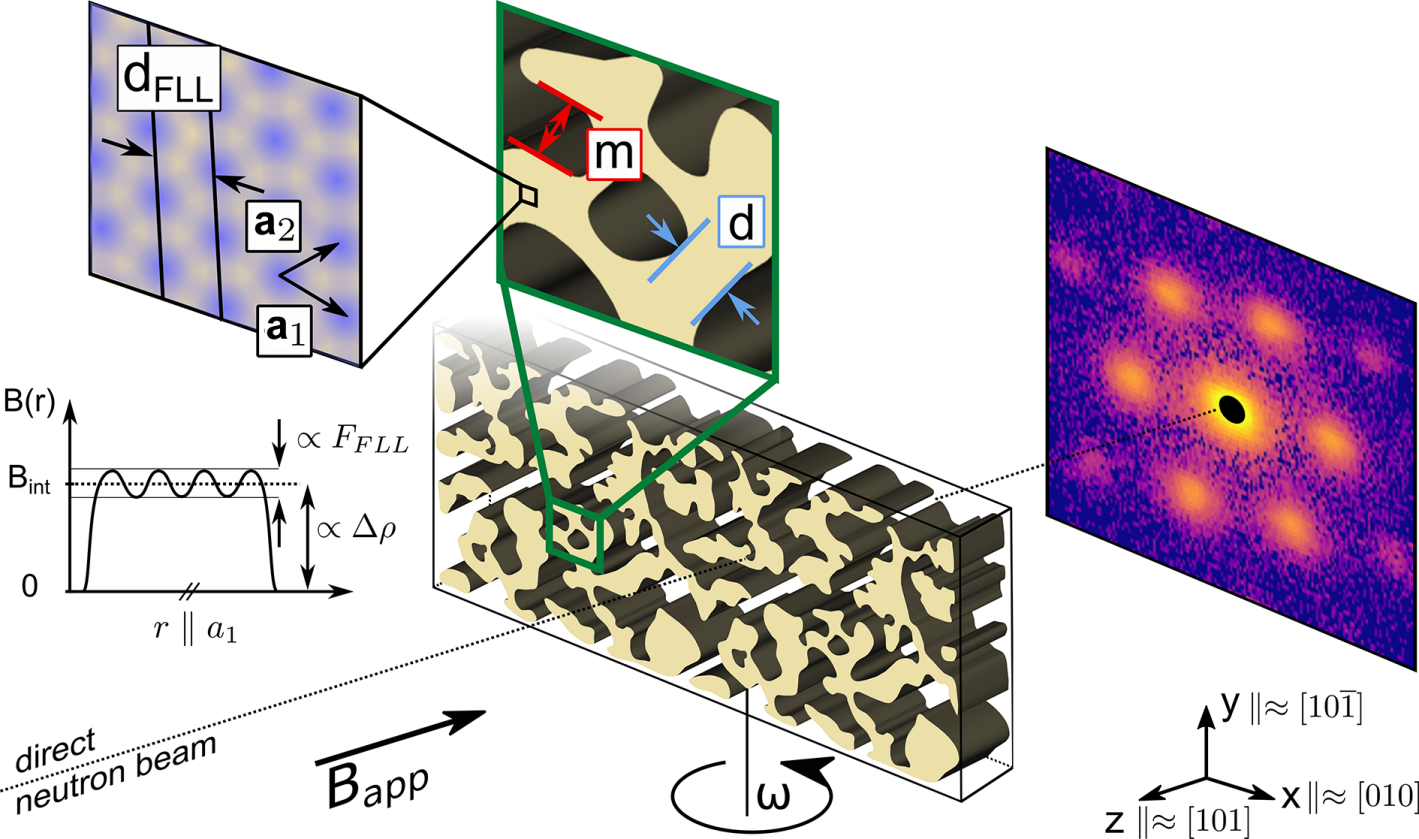
Schematic of the scattering geometry. The applied magnetic field **B**_app_ is aligned parallel to the incoming neutron beam. The scattered neutrons are recorded using a position-sensitive 2D detector placed behind the sample. The two FLL vectors **a**_*i*_, the interplanar FLL distance *d*_FLL_, the FLL domain size *d* and the Meissner state domain size *m* are marked by arrows in the zoomed-in schematics of the insets. A schematic drawing of the magnetic field distribution *B*(*r*) along the direction of **a**_1_ is also included. The magnetic scattering length density contrast Δρ is determined by the difference in magnetic field between mixed state domains (internal magnetic field **B**_int_) and Meissner state domains (zero internal field) and connected to the IMS scattering. The FLL form factor amplitude *F*_FLL_ connected to the Bragg peak scattering results from the close to sinusoidal field variation inside the mixed state domains.

For superconductors on the verge between type I and type II, for example in niobium, we find, below a certain applied field, a coexistence of the FLL interspersed with the Meissner state separated on the micrometre length scale, as schematically depicted in Fig. 1. The resulting domain structure is most commonly known as the intermediate mixed state (IMS) (Träuble & Essmann, 1967 ▸; Krägeloh, 1970 ▸). Since its first observation (Träuble & Essmann, 1967 ▸), the IMS and similar domain structures have been studied in a multitude of superconducting systems (Christen *et al.*, 1980 ▸; Ge *et al.*, 2014 ▸; Vinnikov *et al.*, 2019 ▸) and have recently been rediscovered in both theory and experiment (Ooi *et al.*, 2021 ▸; Brems *et al.*, 2022 ▸; Vagov *et al.*, 2023 ▸; Backs *et al.*, 2023 ▸). While the exact theoretical explanation depends on the superconducting system and is still an active field in theory, in general, the domain formation can be understood as a result of an additional attractive component in the conventional mutual repulsive flux line interaction leading to a locked-in lattice spacing (Brandt & Das, 2011 ▸; Babaev & Silaev, 2013 ▸).

Besides being an interesting topic in superconductivity, the IMS also represents an ideal model system for universal domain physics: it exhibits typical domain shapes such as tubular, laminar and labyrinth-like (Krägeloh, 1970 ▸) which are also observed in other physical, chemical and biological systems (Seul & Andelman, 1995 ▸). This renders the IMS a remarkable model system, as not only the inter flux line spacing of the FLL but also the length scale of the domain structure can be easily tuned via changing the magnetic field and temperature (Backs *et al.*, 2019 ▸).

SANS is routinely used to derive characteristic superconducting parameters from the FLL SANS pattern such as the London penetration depth λ_L_ and Ginzburg Landau coherence length ξ_GL_ (Eskildsen, 2011 ▸), and can also shed light on the superconducting pairing mechanism (Eskildsen, 2011 ▸; Campillo *et al.*, 2021 ▸). One of its main advantages is the use of 2D position-sensitive detectors, allowing for an easy extraction of orientation-dependent structural information. Especially in the case of the FLL, seeing the full 2D picture of the Bragg peak scattering is invaluable for a complete understanding of the FLL structure (Mühlbauer *et al.*, 2019 ▸). However, SANS would struggle to capture lengths much larger than 1 µm as found, for example, in the domain structure of the IMS.

An upper limit in SANS of *d* = 4 µm for a large sample of 15 mm was obtained on D11 located at the Institut Laue–Langevin (ILL) via the combined use of neutron focusing lenses with a special instrument configuration countering the effects of gravity (Cubitt *et al.*, 2011 ▸). For sample sizes of the order of a few millimetres, this upper limit is closer to 1 µm. Techniques such as ultra-small-angle neutron scattering using interferometry (USANS) can extend the accessible length scales to approximately 30 µm (Hainbuchner *et al.*, 2002 ▸). However, this comes at the cost of long counting times due to the use of multiple crystal reflections for the monochromator and analyzer. Additionally, USANS is not able to capture a possible orientational dependence of the scattering pattern due to the 1D nature of the technique.

In the specific case of the IMS domain structure, USANS can give direct access to the average size of the domains. However, the need for an additional sample environment (magnet and cryostat) increases the counting time even further. The lack of orientational information on the scattering pattern persists, adding to the disadvantages of USANS for the study of the IMS. It would be a huge advantage if we could extend the accessible length scales of conventional SANS to cover both the lattice spacing and the micrometre length scale of the IMS, thus giving access to the entire system.

Here we present a novel approach to further push the limits and extract large real-space length scales from the modified Porod analysis of SANS data. Porod scattering (Porod, 1982 ▸) originates from the scattering of a random arrangement of flat, smooth surfaces. The isotropic scattering can be described by a simple power law of the form *q*^−4^, with the modulus of the wavevector transfer *q* defined as 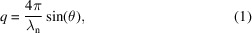
where the neutron wavelength is λ_n_ and 2θ is the angle between the incoming and scattered neutron. Conventional Porod scattering can be described by 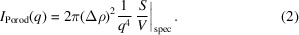
The intensity as a function of *q* is determined only by the scattering contrast Δρ and the quantity 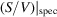, known as the specific surface area. 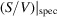 is equivalent to the number density of scatterers per unit volume multiplied by the surface area of a single scatterer and has the unit of an inverse length. We show that, in the special case of an aligned system, such as the IMS, the inverse of 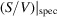 is directly related to a specific length scale of the domain structure. Via this method, we extract the characteristic length scales of up to 40 µm of the domain structure found in the IMS. Our results are consistent with previous attempts to determine this length scale from USANS (Reimann *et al.*, 2017 ▸), Landau’s theory of domains in superconductors (Landau, 1937 ▸) and the well established method of deriving correlation lengths from Bragg peak widths (Cubitt *et al.*, 1992 ▸; Cubitt, 1994 ▸; Grigoriev *et al.*, 2010 ▸) that can be connected to the finite size of the FLL domains.

The paper is structured as follows. We first present the experimental setup and experiment geometry in Section 2. In Section 3, we then focus on the SANS data analysis method to determine quantitative information from the FLL Bragg peaks and the power-law scattering of the domain structure via the modified Porod analysis. The results from our analysis are presented and discussed in Section 4 and Section 5, where we additionally compare our results with previous findings and Landau’s theory of domains in superconductors. Finally, we summarize our main findings in Section 6.

## 2. Experimental setup

The SANS measurements were performed using the small-angle neutron diffractometer D33 at the ILL (Dewhurst *et al.*, 2016 ▸). The standard FLL SANS experiment geometry is depicted in Fig. 1 ▸, showing the orientation of the sample with its crystallographic orientation, the direction of the applied magnetic field **B**_app_ and the coordinate system used from now on.

We used a large prism-shaped high-purity Nb single-crystal sample (*l* × *w* × *t* = 13 × 3.8 × 1 mm) in this study. The sample was cut from the same Nb single crystal obtained from Heraeus used in previous studies on the IMS (Reimann *et al.*, 2017 ▸; Backs *et al.*, 2019 ▸; Brems *et al.*, 2022 ▸). A residual resistivity ratio of RRR ≃ 390 (Brems *et al.*, 2022 ▸) and a superconducting transition temperature in zero field *T*_c_ = 9.25 K obtained from transport and AC magnetic susceptibility measurements, respectively, were measured using a smaller sample cut from the same single crystal. The value of *T*_c_ agrees well with the literature value of Nb (Stromberg, 1965 ▸) and confirms the high degree of purity. The [101] and [010] crystallographic directions were measured to be parallel to the large sample face surface normal and parallel to the long sample dimension, respectively, within a few degrees using an X-ray diffractometer, resulting in the orientation as shown in the coordinate system of Fig. 1 ▸. The sample was positioned in the neutron beam with the long sample dimension parallel to the *x* axis and the large sample face aligned perpendicular to the magnetic field, resulting in a large demagnetizing factor and large phase space of the IMS in the field temperature phase diagram.

The magnetic field was initially aligned parallel to the incoming neutron beam, but the ensemble of the cryostat with the sample inside the magnet could be rotated by an angle ω around the *y* axis, perpendicular to the neutron beam. A maximum collimation length of *D*_col_ = 12.8 m, sample–detector distance *D*_det_ = 13 m and neutron wavelength λ_n_ = 14 Å with a full width at half-maximum (FWHM) wavelength spread 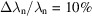 were used. The beam size was defined by a neutron absorbing source aperture of diameter *d*_source_ = 10 mm and a rectangular neutron absorbing sample aperture of *a*_*x*_ × *a*_*y*_ = 5 × 1.5 mm illuminating the central part of the sample. This resulted in an effective sample volume in the beam of *V* = 5 × 1.5 × 1 mm. The magnetic field and cryogenic temperatures were supplied via a dry high-*T*_c_ superconducting magnet in combination with an Orange-type cryostat. All measurements were performed at *T* = 4 K following a field-cool (FC) measurement procedure from *T* = 10 K and were corrected for a high-temperature background measured in zero field at *T* = 10 K above the superconducting transition temperate *T*_c_. For each measured field, we scanned the angle ω in the range of −1° to 1° with a step size of Δω = 0.05°, referred to as a rocking scan.

## 3. SANS data reduction and analysis

**Figure 2:**
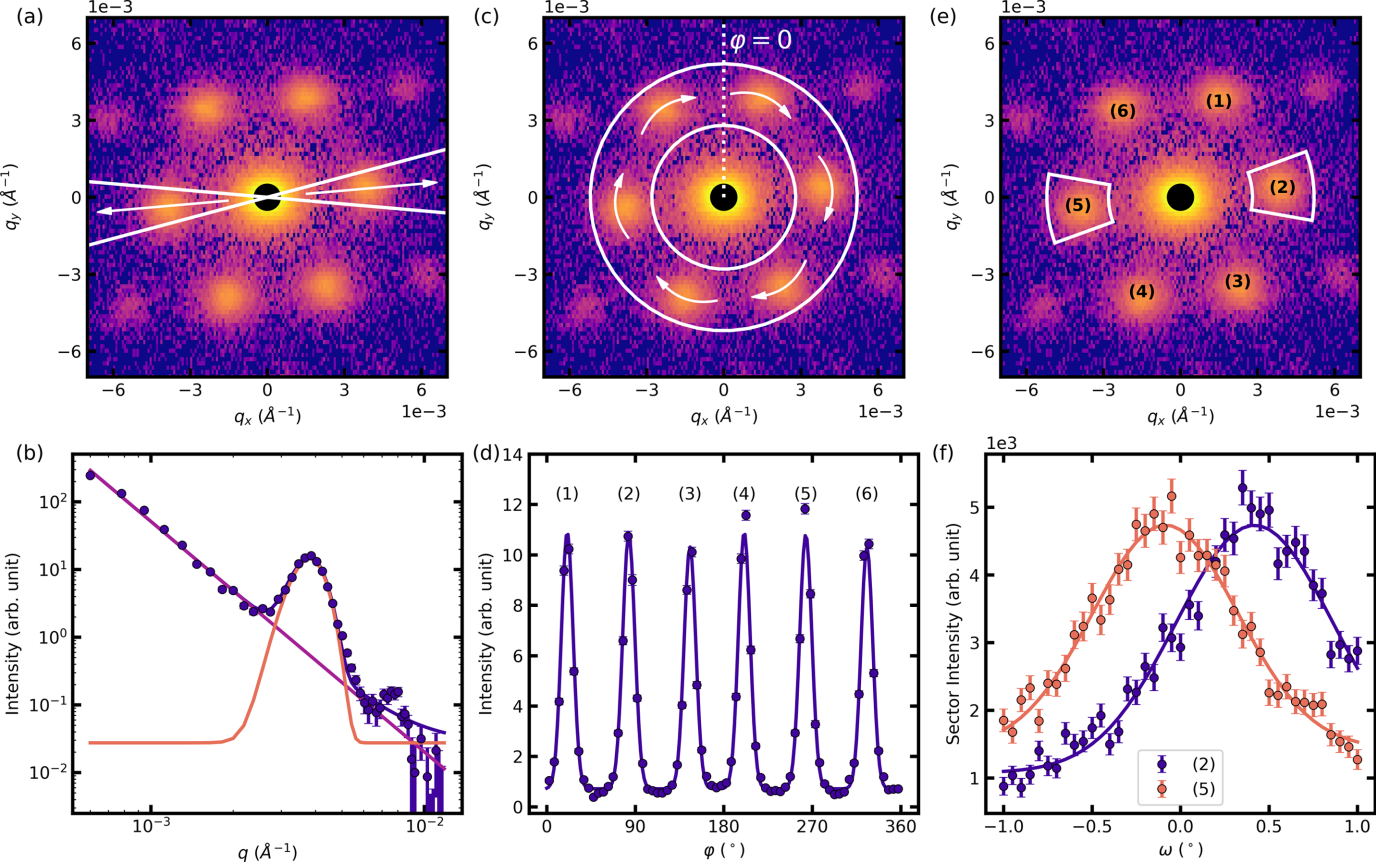
From 2D scattering data to 1D reduced data on the example of data measured in *B*_app_ = 20 mT and *T* = 4 K. The top panels show the background-corrected 2D SANS detector image of the sum of the rocking scan. The columns represent the extraction of the radially averaged data (*a*), (*b*), azimuthally averaged data (*c*), (*d*) and rocking curves (*e*), (*f*) from the background-corrected 2D data to fit the radial, tangential and longitudinal Bragg peak widths, respectively.

The different length scales found in the IMS call for a careful analysis of the SANS pattern that we will develop in the following. Before we focus on extracting physical quantities from the scattering data, we turn our attention to an example of typical 2D SANS data of our system [shown in Fig. 2 ▸(*a*)] to identify the two distinct contributions: (i) the FLL inside the mixed state domains with an interplanar FLL distance *d*_FLL_ of 100–200 nm results in well defined Bragg peak scattering and (ii) the domain structure consisting of mixed state and Meissner state domains separated on the micrometre length scale leads to diffuse scattering in the low-*q* regime found in the vicinity of the blacked-out direct beam. The *I*-versus-*q* curve shown in Fig. 2 ▸(*b*) obtained by radially averaging the intensity inside the white sectors of Fig. 2 ▸(*a*) allows us to better understand the qualitative difference in the two scattering contributions: We can extract both the peak position of the Bragg peak scattering and the three reciprocal widths in the radial, tangential and longitudinal directions. The diffuse scattering, on the other hand, is best described using a power law. The data set consists of 2D SANS detector images recorded at different rocking angles ω. After normalization, calibration and background subtraction, the data set is reduced to 1D scattering curves in order to perform a quantitative data analysis of the two scattering contributions. In the first part of this section, we outline the principal steps of our data reduction workflow. In the second part, we focus on the extraction of length scales and quantities from the reduced data, starting with the Bragg peak scattering connected to the FLL. In the third part, we turn our attention to a detailed analysis of the low-*q* power-law scattering stemming from the domain structure.

### 3.1. SANS data reduction

Standard data correction is applied using the SANS data reduction tool *GRASP* (Dewhurst, 2023 ▸) to calculate the corrected scattering data *I*_corr_, 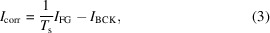
with the foreground data *I*_FG_, the background data *I*_BCK_ and the relative beam attenuation of the sample expressed as a transmission *T*_s_. The additional beam attenuation in the foreground data with respect to the background data is caused by the magnetic scattering in the superconducting state. Strictly speaking, the data correction scheme presented in equation (3) is only valid for beam attenuation due to absorption (Dewhurst, 2023 ▸), but it can be used to perform background correction on neutron scattering data of the IMS (Reimann *et al.*, 2015 ▸).

In the analysis of the low-*q* power-law scattering, where we limit the analysis to the rocking angle ω = 0, *T*_s_ is calculated for each measured field as the ratio of the intensity of the transmitted direct beam of the foreground and the background data, both at ω = 0. In the analysis of the Bragg peak scattering, where only the position and width of the Bragg peaks are relevant, *T*_s_ is set to 1. After background subtraction, *I*_corr_ only contains the scattering due to magnetic structures. Absolute scattering intensities are obtained via the standard SANS data reduction dividing the intensity by the illuminated sample volume, the direct beam flux and the pixel solid angle.

For radially and azimuthally averaged data (see Fig. 2 ▸ for examples) the scattering intensity in each pixel is summed over the entire range of rocking angles. For the radially averaged data used in the analysis of the low-*q* power-law scattering we only consider ω = 0. The intensity is then represented in polar coordinates as a function of the modulus of the wavevector transfer *q* and azimuthal angle φ to yield *I*(*q*, φ). The azimuthal angle φ is defined on the detector plane with respect to the *q*_*y*_ axis as shown in Fig. 2 ▸(*c*). For radially averaged data we integrate over φ to obtain *I*(*q*) [see Figs. 2 ▸(*a*), 2 ▸(*b*)], whereas for azimuthally averaged data we integrate over *q* to get *I*(φ) [see Figs. 2 ▸(*c*), 2 ▸(*d*)]. User-defined masks, such as sectors around Bragg peaks [see Fig. 2 ▸(*a*)], can be applied to adapt the area of integration.

For the rocking curves, the scattering data are not summed over rocking angles. Instead, the intensity inside a region of interest given by a user-defined mask is summed for each rocking angle ω to obtain *I*(ω) [see Figs. 2 ▸(*e*), 2 ▸(*f*)].

### 3.2. Bragg peak scattering of the FLL

**Figure 3:**
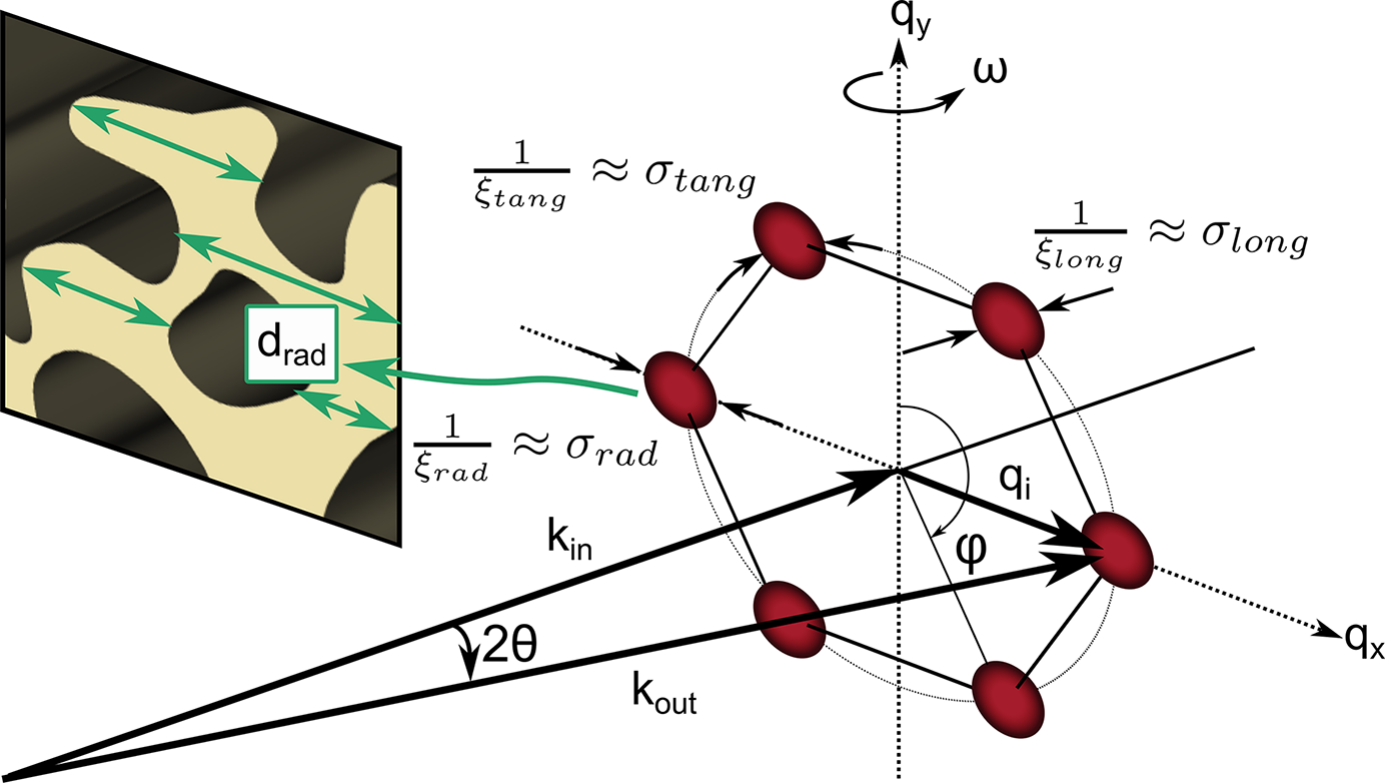
Schematic drawing of the intrinsic widths σ_*i*_ of the Bragg peaks in the three reciprocal-space directions and the corresponding real-space correlation lengths ξ_*i*_. The inset shows schematically the projected length *d*_rad_ along the direction parallel to 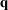 of the horizontal Bragg peaks contributing to the Bragg peak width σ_rad_ via a finite size broadening.

We now focus on quantities extracted from the Bragg peak scattering characterized by the positions and widths of the six first-order Bragg peaks, as schematically depicted in Fig. 3 ▸.

#### 3.2.1. Internal magnetic field *B*_int_

Each Bragg peak has a well defined position **q**_*i*_ = (*q*_*i*_, φ_*i*_) given by the modulus of the wavevector transfer *q*_*i*_ and the azimuthal angle φ_*i*_. The value of the local internal magnetic field, *B*_int_, is calculated using the position **q**_*i*_ of two adjacent first-order Bragg peaks according to 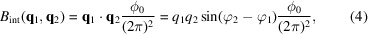
with the magnetic flux quantum ϕ_0_ = *h*/2*e* of a flux line, where *e* is the elementary charge and *h* is the Planck constant.

The Bragg peak positions in *q* are determined by fitting a function consisting of the sum of a power law ∝ *q*^−4^ and a Gaussian function, with peak position *q*_0_, Gaussian width σ expressed as a root mean square (r.m.s.), intensity *I*_0_ and zero offset *y*_0_, to the radially averaged intensity inside sectors encompassing a pair of Bragg peaks shown on the example of the horizontal Bragg peaks in Fig. 2 ▸(*a*). By including the power-law scattering, we ensure that the underlying IMS scattering is not leading to an overestimation towards larger *q* values of the Bragg peak position. The peak positions in azimuthal angle φ are extracted by fitting a sum of six Gaussian functions with common zero offset *y*_0_ to the azimuthally averaged intensity inside the sector shown between white circles in Fig. 2 ▸(*c*). We reduce the number of fit parameters by making use of the symmetry of the system: two opposing Bragg peaks have the same r.m.s. width σ, intensity *I*_0_ and peak position φ_0_ that is shifted by π.

#### 3.2.2. Correlation lengths

In standard crystal diffraction, the finite size of the crystal contributes to the width of the measured Bragg peaks, known as finite size broadening. The same concept can be applied to SANS Bragg peaks of large-scale crystalline structures such as the FLL (Cubitt, 1994 ▸) or nanopore lattices (Grigoriev *et al.*, 2010 ▸) to quantify the size of the well ordered lattice structure. The volume defined by the extent of the well ordered FLL can be expressed using three correlation lengths ξ_*i*_ inversely connected to the intrinsic radial σ_rad_, tangential σ_tang_ and longitudinal σ_long_ width of the FLL Bragg peaks in reciprocal space, schematically depicted in Fig. 3 ▸. The measured widths of the Bragg peaks in the three reciprocal-space directions are a result of the convolution of the different broadening effects due to the size and perfection of the well ordered FLL, the sample volume, and the instrumental resolution defining a coherence volume. Whichever is the smallest dominates the measured Bragg peak widths σ_*i*,m_.

The intrinsic Bragg peak widths are calculated from the respective measured widths σ_*i*,m_ by taking into account the instrumental resolution and their mutual contributions according to the method originally presented by Cubitt (1994 ▸) and extended by Campillo *et al.* (2022 ▸). In the calculation the intrinsic Bragg peak widths in the reciprocal space are expressed as the intrinsic mosaic spread along the field direction σ_*b*_ in radians, the intrinsic tangential spread σ_t_ in radians on the detector plane and the relative spread of interplanar distances 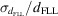. The intrinsic r.m.s. Bragg peak widths σ_*i*_ in reciprocal-space units are then calculated from these quantities given as r.m.s. widths according to 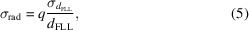

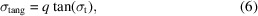

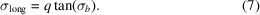


Converting the intrinsic widths of the Bragg peaks in reciprocal space to meaningful correlation lengths in real space is prone to subjectivity, as can be seen from the different forms used in the literature (Yaron *et al.*, 1994 ▸, 1995 ▸; Backs *et al.*, 2019 ▸; Grigoriev *et al.*, 2010 ▸; Eskildsen, 2011 ▸). A full discussion on how to properly define and extract correlation lengths is not the focus of this work; however, we outline some common considerations and justify our choice. In the conventional mixed state, where the correlation length serves to quantify the FLL perfection, the correlation length is often defined as the inverse of the r.m.s. Bragg peak width σ in reciprocal space, which results in ξ = 1/σ (Eskildsen, 2011 ▸). This is obtained from the width of the Fourier transform of a Gaussian distribution of r.m.s. width σ, which again is a Gaussian distribution of r.m.s. width ξ = 1/σ. The real-space length scale is the envelope over FLL positions where the lattice is still in phase. Sometimes, factors of 2 appear to account for a different peak shape (Yaron *et al.*, 1994 ▸, 1995 ▸). In the case of the IMS, we use the correlation length to extract the actual size of the mixed state domains. Note that the peak broadening is not solely caused by the distribution of the different broadening effects such as a distribution of interplanar lattice spacing *d*_FLL_, but rather a result of a top hat function convolved with the FLL structure representing the finite size of the mixed state domains. While observed on a different length scale, this is analogous to the Bragg peak broadening encountered in powder diffraction experiments. The Scherrer equation (Scherrer, 1918 ▸) can be used to determine the crystallite size τ in the direction perpendicular to the lattice planes from the Bragg peak width Δ(2θ) at the Bragg angle θ according to 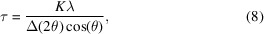
where λ is the wavelength of the used radiation, *K* is a dimensionless factor close to unity connected to the shape of the crystallite and Δ(2θ) is expressed as an FWHM in radians. Using the small-angle approximation and the definition of the wavevector transfer *q* ≃ 2π2θ/λ, this can be rewritten as 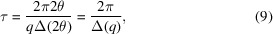
where we set *K* = 1 for simplicity and in the last equation the relation Δ(*q*)/*q* = Δ(2θ)/2θ was used with Δ(*q*) expressed as an FWHM. A definition of the correlation length according to equation (9) seems more appropriate to quantify the size of the mixed state domains in the IMS domains. It is also used outside the field range of the IMS to avoid discontinuities due to a change of the definition of the correlation length. As the Bragg peak widths are extracted as r.m.s. widths, the real-space correlation lengths are then obtained as ξ_*i*_ = 2π/*k*σ_*i*_, with the scaling factor 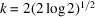 converting r.m.s. widths σ_*i*_ to their FWHM equivalent.

As we only performed rocking scans around the vertical *x* axis, we can only fully extract the FLL correlation lengths of the equivalent spots (2) and (5) marked in Fig. 2 ▸(*e*).

The measured radial and tangential widths were determined from the same fit used to extract the Bragg peak positions in *q* and φ. From the rocking curves shown in Fig. 2 ▸(*f*) of the intensity inside the sectors marked in white in Fig. 2 ▸(*e*), we determine the r.m.s. width of the rocking curve by fitting a Gaussian function.

### 3.3. Porod scattering of the domain structure

Having discussed the extraction of physical quantities from the Bragg peak scattering, we now focus on the *q*^−4^ power-law scattering observed at low *q* values. The Porod law (Porod, 1982 ▸) can be used to describe the *q*^−4^ power-law scattering of scattering objects with well defined surfaces that are either in a vacuum or surrounded by a second phase. It is used to connect the specific surface area 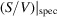 of the scattering objects to their macroscopic scattering intensity according to 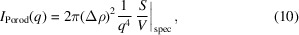
with the scattering length density contrast Δρ = ρ_1_ − ρ_2_ given as the difference between the scattering length densities of the two phases. Δρ reduces to ρ, the scattering length density of the scattering particles, in the absence of a second phase.

Equation (10) is derived using the angular average of 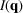 over all possible directions of 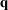 and is valid only in the case of spherical symmetric scattering objects (Feigin & Svergun, 1987 ▸), where the scattering intensity depends only on the magnitude *q* and not the direction given by its unit vector 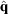. It has been adapted to anisotropic particulate samples possessing smooth boundaries (*i.e.* with no sharp edges and/or vertices) and strictly convex particles.^1^ The scattering intensity shows the same asymptotic *q*^−4^ behavior (Ciccariello *et al.*, 2000 ▸) according to 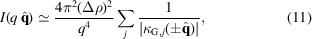
where 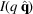 stands for the *q*-dependent intensity along a fixed direction 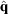 and κ_G, *j*_ denotes the Gaussian curvature of the *j*th scattering particle. The notation 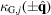 ensures that only the particle curvatures with their surface normal parallel or antiparallel to the unit vector 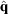 enter in the calculation of the scattering intensity in the direction of 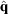. As a result, the measured scattering intensity is higher for directions where the Gaussian curvature is smaller, which is equivalent to directions where the surface of the particles is close to flat.

In an alternative formulation, closely reflecting the morphology of the IMS, the Porod scattering of anisotropic two-phase systems can be rewritten in a more suitable manner. Using the angular distribution of the surface normals between the two phases in the sample 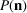, the power-law scattering can be modeled according to 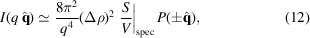
where 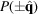 stands for the angular distribution 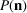 evaluated for directions parallel/antiparallel to 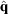 (Onuki, 1992 ▸). We can recover the conventional Porod law of equation (10) by inserting the isotropic case 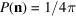 (Onuki, 1992 ▸). Comparing the conventional Porod law in equation (10) with equation (12), the correct value of 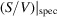 cannot be obtained using the conventional Porod law in the case of samples with anisotropic particles/surface normals. In our case, the domain structure is aligned with its surface normals close to orthogonal to the incoming neutron beam, leading to a massive overestimation of 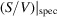 when extracted using equation (10). A correct determination of 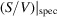 is only possible taking into account 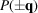. In the case of the IMS, where the surface normals are close to orthogonal to the incoming neutron beam, this reduces to a simple scaling factor α_orient_.

In the following we connect the rocking-curve width of the low-*q* IMS intensity to α_orient_ to extract the corrected value of 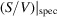. In a second step the corrected value will be connected to a characteristic length scale of the domain structure.

#### 3.3.1. Correction factor α_orient_ for highly oriented structures

**Figure 4:**
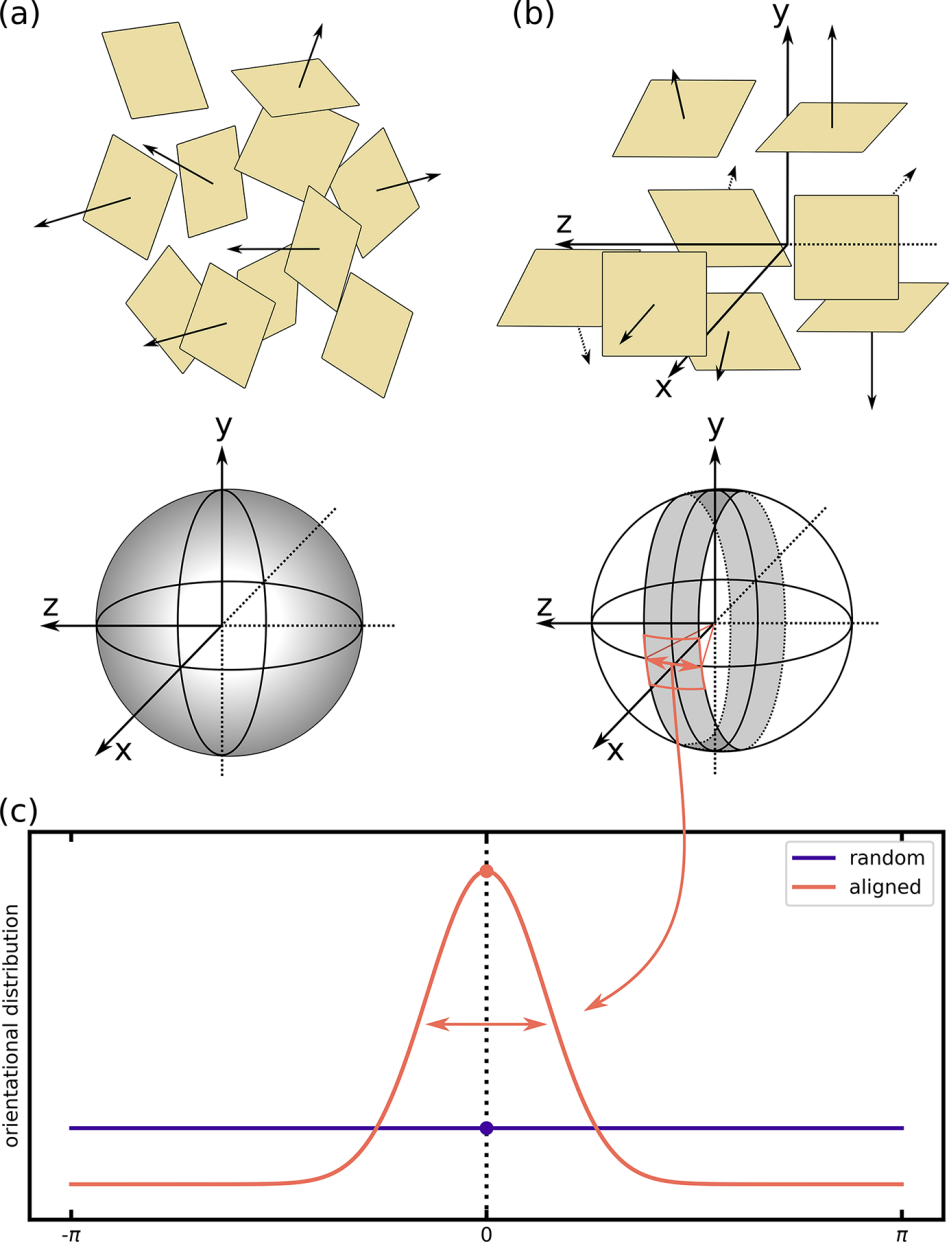
Schematic representation of the surface normals in a sample with randomly oriented scattering objects (*a*) versus a sample with aligned surfaces such as the studied domain structure (*b*). (*c*) illustrates the distribution of the random (purple line) and aligned case (orange line), which for radially averaged data is just a 1D curve. The distribution of the aligned case is extracted from the IMS rocking curves shown in Fig. 5 ▸(*d*). The correction factor α_orient_ is given as the ratio of the constant value of the flat distribution and the peak value of the aligned distribution marked by a purple and orange solid dot, respectively. Note that the width of the shown distribution is exaggerated for illustrative purposes and not to scale.

Without loss of generality, the surfaces of the scattering particles making up the sample can be considered as square-shaped platelets. In the random case, they are isotropically oriented in space, as schematically depicted in Fig. 4 ▸(*a*). The distribution of their surface normals can be best visualized by the surface of the unit sphere.

In the case of perfectly aligned platelets, the vectors describing the surface normals are found in a plane orthogonal to the neutron beam, as schematically depicted in Fig. 4 ▸(*b*). In the presence of some finite degree of imperfection, the surface normals are distributed with a finite spread around that plane. The spread can be modeled using a Gaussian distribution of width σ, schematically represented by the gray ribbon on the unit sphere in Fig. 4 ▸(*b*).

The Porod law given in equation (10) describes the scattering intensity as a function of the modulus of the wavevector transfer *q*, averaged over all possible directions of 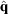. As the low-*q* power-law scattering of the IMS is isotropic on the detector plane, the calculation of the scaling factor reduces to a 1D problem. The uniform probability density distribution over 2π is now compressed into the small ribbon best described by a distribution of r.m.s. width σ, as schematically shown in Fig. 4 ▸(*c*).

**Figure 5:**
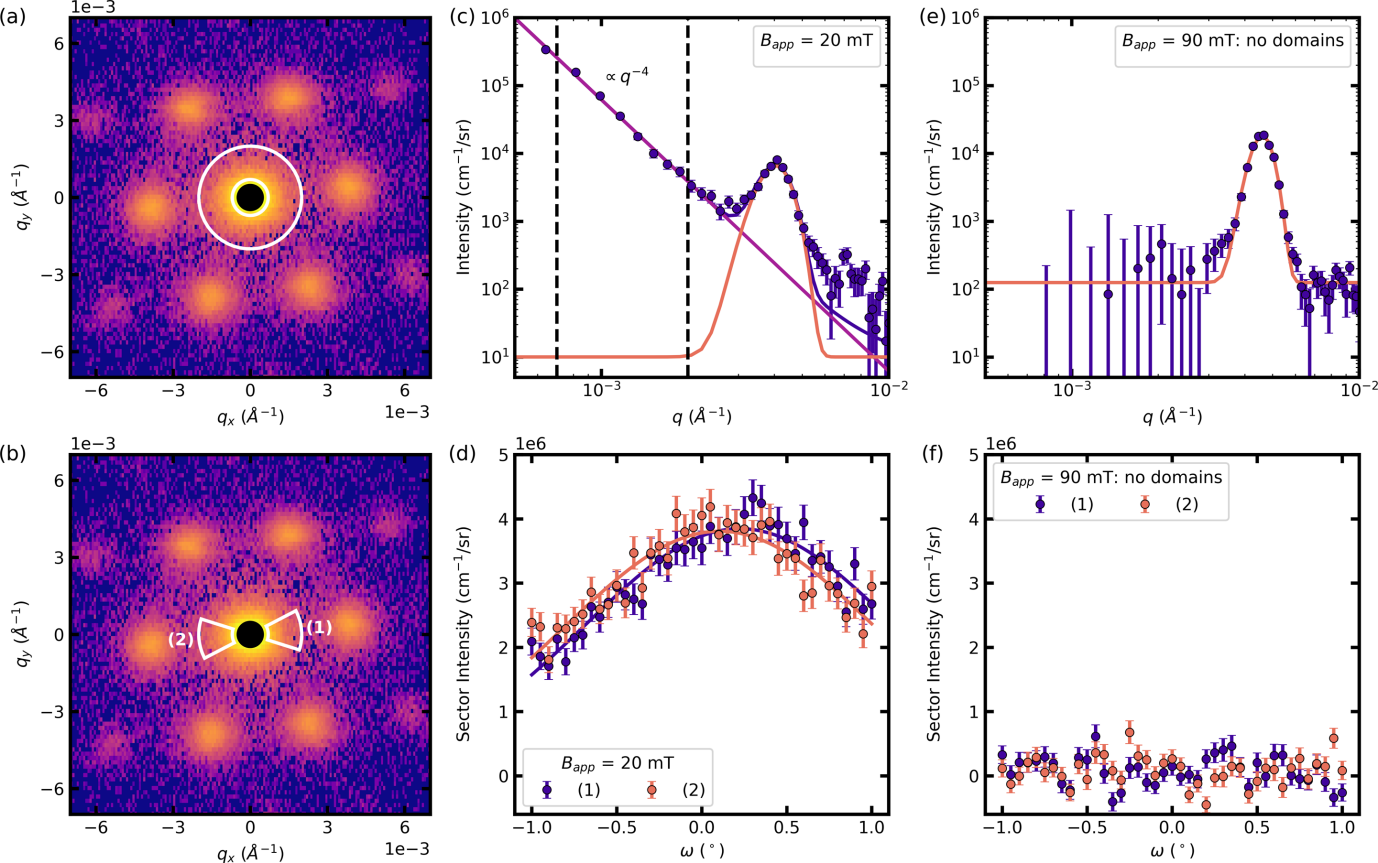
Examples of scattering data to extract the low-*q* scattering connected to the specific surface area of the domain structure: (*a*), (*b*) 2D detector images of the scattering pattern in *B*_app_ = 20 mT, with the definition of the low-*q* sector (*a*) for the extraction of the radially averaged *I*-versus-*q* plot [shown in (*c*)] and the IMS rocking scan sector (*b*) for the extraction of the IMS rocking curve [shown in (*d*)]. (*c*) The fit of the power law to extract the IMS intensity is constrained to the data in between the dashed vertical lines. (*d*) The rocking curve of the IMS intensity within the sector shown in (*b*) is fitted with a Gaussian function to extract its width. Equivalent 1D plots for *B*_app_ = 90 mT where no domain structure is present: no low-*q* power-law scattering is observed in the *I*-versus-*q* plot (*e*) and the rocking curve is flat around zero intensity (*f*).

Ignoring this alignment would result in an overestimation of 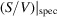. We define the factor α_orient_ as the ratio of the isotropic Porod scattering and the aligned case. Therefore, the corrected specific surface area 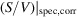 extracted from the modified Porod law for domain structures aligned along the beam direction reads 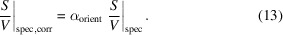
α_orient_ is given as the ratio of the constant value of a normalized uniform distribution on the unit circle and the peak value of a normalized Gaussian of r.m.s. width σ measured in radians, according to 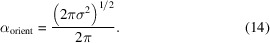
α_orient_ is calculated using the IMS rocking-curve width σ_IMS_ as an estimate of the width of the distribution of surface normals perpendicular to the field direction. Fig. 5 ▸(*d*) shows an example of the IMS rocking curves measured at *B*_app_ = 20 mT. The region of interest for the summation is represented by the white sectors shown in Fig. 5 ▸(*b*). The rocking curves are fitted with a Gaussian function, shown as solid lines, to obtain the r.m.s. width σ_IMS_ given as the average of the rocking-curve widths of the intensity inside sectors (1) and (2).

#### 3.3.2. Extracting length scales from (*S*/*V*)|_spec,corr_

**Figure 6:**
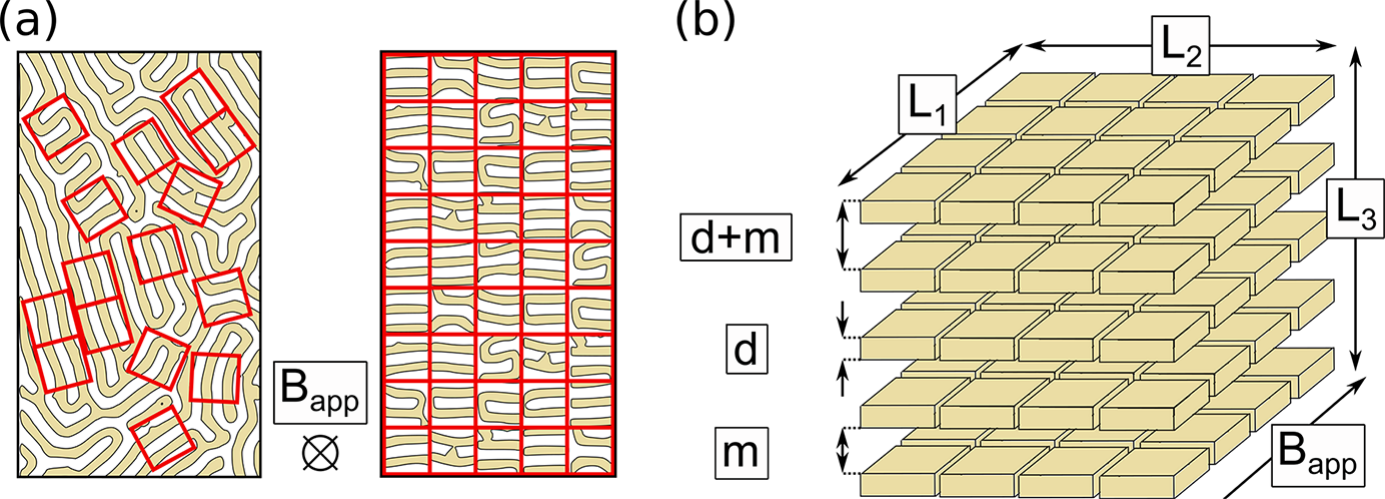
Schematics of the virtual reorientation of randomly oriented domains to aligned planes. The direction of the applied magnetic field **B**_app_ is indicated by the black cross and the black arrow in panels (*a*) and (*b*), respectively. The superconducting domain structure in panel (*a*) is adapted from Brandt & Essmann (1987 ▸), reproduced with permission of Wiley.

Having derived the correction factor α_orient_, we will now turn to extracting a characteristic length scale from 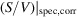 by virtually rearranging the domain structure found in the IMS.


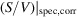
 is the total interface between the two phases divided by the sample volume *V*. The domain structure can be imagined as small areas where the phase boundaries are parallel to each other, as schematically depicted in Fig. 6 ▸(*a*). This results in a system of layers of mixed state domains of thickness *d* separated by Meissner state domains of thickness *m*. The thicknesses *m* and *d* represent an average over all observed thicknesses present in the system. In Fig. 6 ▸(*b*) a sketch of this system is shown, assuming that the individual layers of the mixed state can be pictured as small platelets of area *l*_1_ × *l*_2_ and thickness *d* separated by a distance given as the thickness *m* of the Meissner state domains. The total number of interfaces *N* is then given as 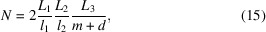
where *L*_1_ × *L*_2_ × *L*_3_ = *V* describes the dimensions and the volume *V* of the sample and the factor 2 stems from the fact that each platelet has two surfaces. Using equation (15), 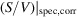 can be connected to the repetition length *m* + *d*,
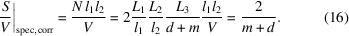
In the special case of a two-phase system with alignment along one direction, 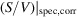 is essentially a length scale. It can be used to extract the field-dependent repetition length *m* + *d* of the IMS.

The only remaining issue before *m* + *d* can be calculated from 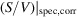 is the determination of the scattering length density contrast Δρ in equation (10).

For scattering patterns with purely magnetic scattering, as shown for example in Fig. 2 ▸(*a*), the scattering stems from the interaction of the neutron’s magnetic moment 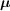 with the magnetic field **B** governed by the Zeemann interaction given as 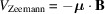. In analogy to the nuclear scattering length density, a magnetic scattering length density contrast can be defined. For the two-phase system consisting of flux-free Meissner state domains and mixed state domains with local internal magnetic field value *B*_int_, Δρ is given as 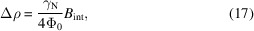
with γ_N_ = 1.91 the ratio between the magnetic moment of the neutron and the nuclear magneton μ_N_ and Φ_0_ the magnetic flux quantum already introduced above.

*B*_int_ is calculated from the Bragg peak positions according to equation (4).

Inserting into equation (17) a typical value of *B*_int_ = 73 mT measured inside the mixed state domains of the domain structure at *T* = 4 K results in a value of Δρ = 1.7 × 10^−7^ Å^−2^.^2^


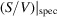
 is obtained by fitting equation (10) with Δρ defined by equation (17) to the radially averaged intensity of the scattering pattern at ω = 0. The fit was constrained to data found in between the white circles shown in Fig. 5 ▸(*a*). This corresponds to the intensity in between the vertical dashed lines shown in Fig. 5 ▸(*c*). 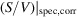 is then calculated according to equation (13) using α_orient_ according to equation (14).

## 4. Results

The *Results* section follows the same structure as the previous section. We first focus on *B*_int_ and ξ_*i*_ extracted from the FLL Bragg peaks. We then turn our attention to 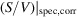 and the repetition length *m* + *d* extracted from the power-law scattering of the domain structure.

**Figure 7:**
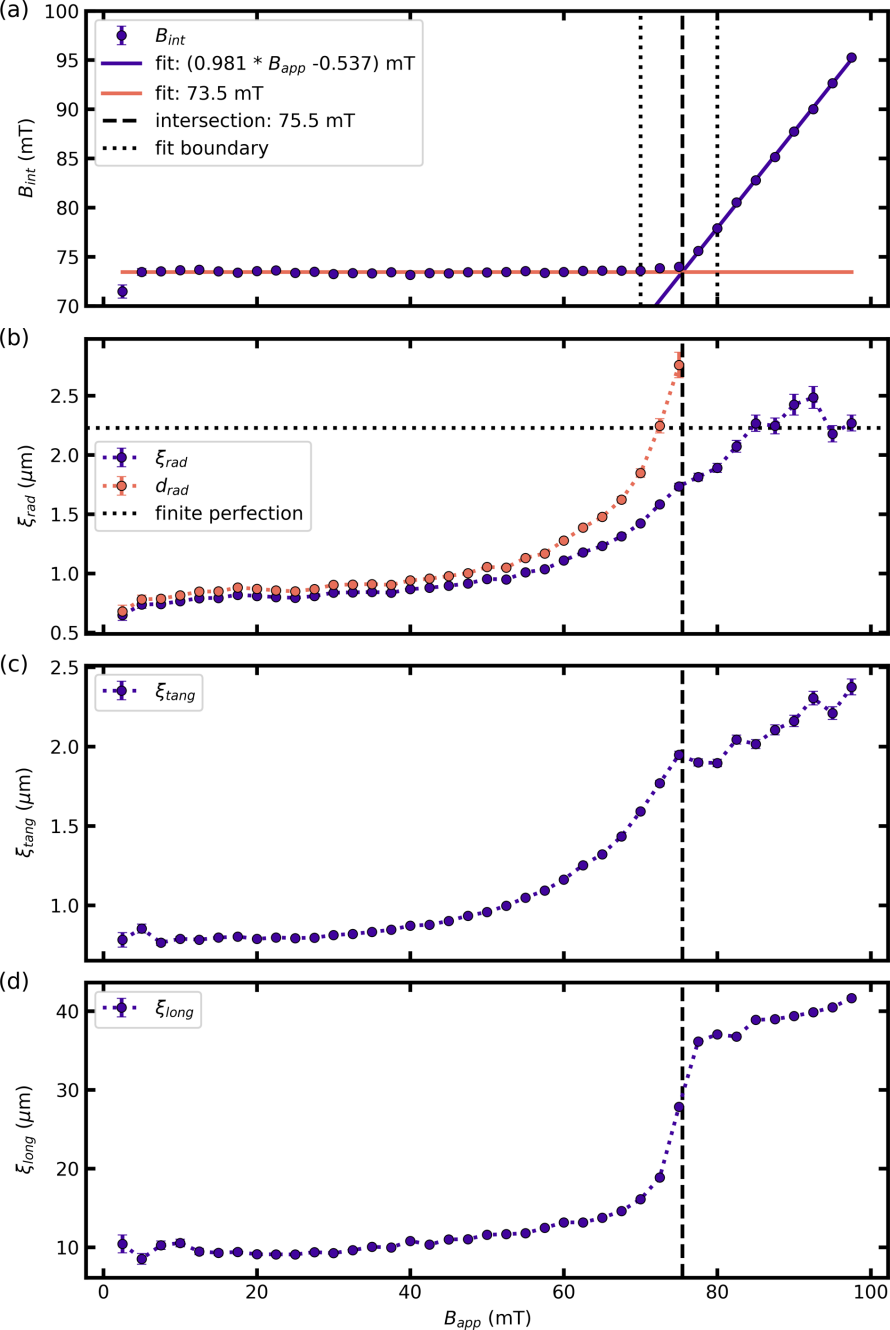
(*a*) Local internal magnetic field *B*_int_ and (*b*)–(*d*) radial, tangential and longitudinal correlation length extracted from the Bragg peak width in the three reciprocal-space directions. (*b*) additionally shows the size of mixed state domains *d*_rad_.

Fig. 7 ▸(*a*) shows the value of the local internal field *B*_int_ inside the mixed state domains as a function of the applied field *B*_app_ obtained from the positions **q**_*i*_ of two adjacent Bragg peaks according to equation (4). We observe two different regimes separated by the value of the applied field *B*_app_ = B* ≃ 75 mT: starting at high fields *B*_int_ decreases linearly with decreasing field down to *B*_app_ = B*. For lower fields, we find a constant value of *B*_int_ = (73.5 ± 0.3) mT with a slight downturn for *B*_app_ = 2.5 mT. The two different regimes can both be fitted using a straight line *B*_int_ = *aB*_app_ + constant, where for the low-field regime we set *a* = 0. We excluded the field range close to the transition marked in between the two dotted vertical lines. The fits are shown as solid purple and orange lines in Fig. 7 ▸(*a*) for *B*_app_ > B* and *B*_app_ < B*, respectively. The line of intersection of the two regimes is obtained from equating the two fits and results in a value of B* = 75.5 mT. It is included as a vertical dashed line in all panels of Fig. 7 ▸.

The two regimes can also be traced via the FLL correlation lengths obtained from the Bragg peak widths in the three reciprocal-space directions. We first describe the correlation lengths ξ_rad_ and ξ_tang_ shown by the purple markers in Figs. 7 ▸(*b*) and 7 ▸(*c*), respectively. Both correlation lengths lie in the plane orthogonal to the applied field. Starting at high applied fields in the conventional mixed state ξ_rad_ is scattered around a constant value ξ_finite_ ≃ 2.2 µm.

For *B*_app_ < B*, ξ_rad_ rapidly decreases and then levels off to a length of ξ_rad_ ≃ 0.7 µm for the lowest applied fields. ξ_tang_ shows a very similar behavior with slight deviations in the high-field regime: in contrast to the scatter around a constant value observed at high applied fields, ξ_tang_ exhibits a slow decrease before it rapidly decreases at field values close to the transition line B*. We again observe a leveling off to a constant value of ξ_tang_ ≃ 0.8 µm. We now describe the field dependence of the correlation length ξ_long_ in the direction of the applied field shown in Fig. 7 ▸(*d*). Over the entire field range, the values of ξ_long_ are larger by more than an order of magnitude than the respective values of ξ_rad_ and ξ_tang_. This is not surprising for the FLL as the flux lines, the building blocks of the FLL, are long structures elongated along the field direction. The high-field value for *B*_app_ > B* is close to 40 µm. At B* we observe the same rapid decrease of the correlation length and a consequent leveling off to a low-field value of ξ_long_ ≃ 10 µm when approaching zero field.

We now turn to the analysis of the low-*q* power-law scattering caused by the domain structure. Using the modification of the Porod analysis presented in Section 3, we extract 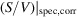 to subsequently calculate the repetition length *m* + *d*.

**Figure 8:**
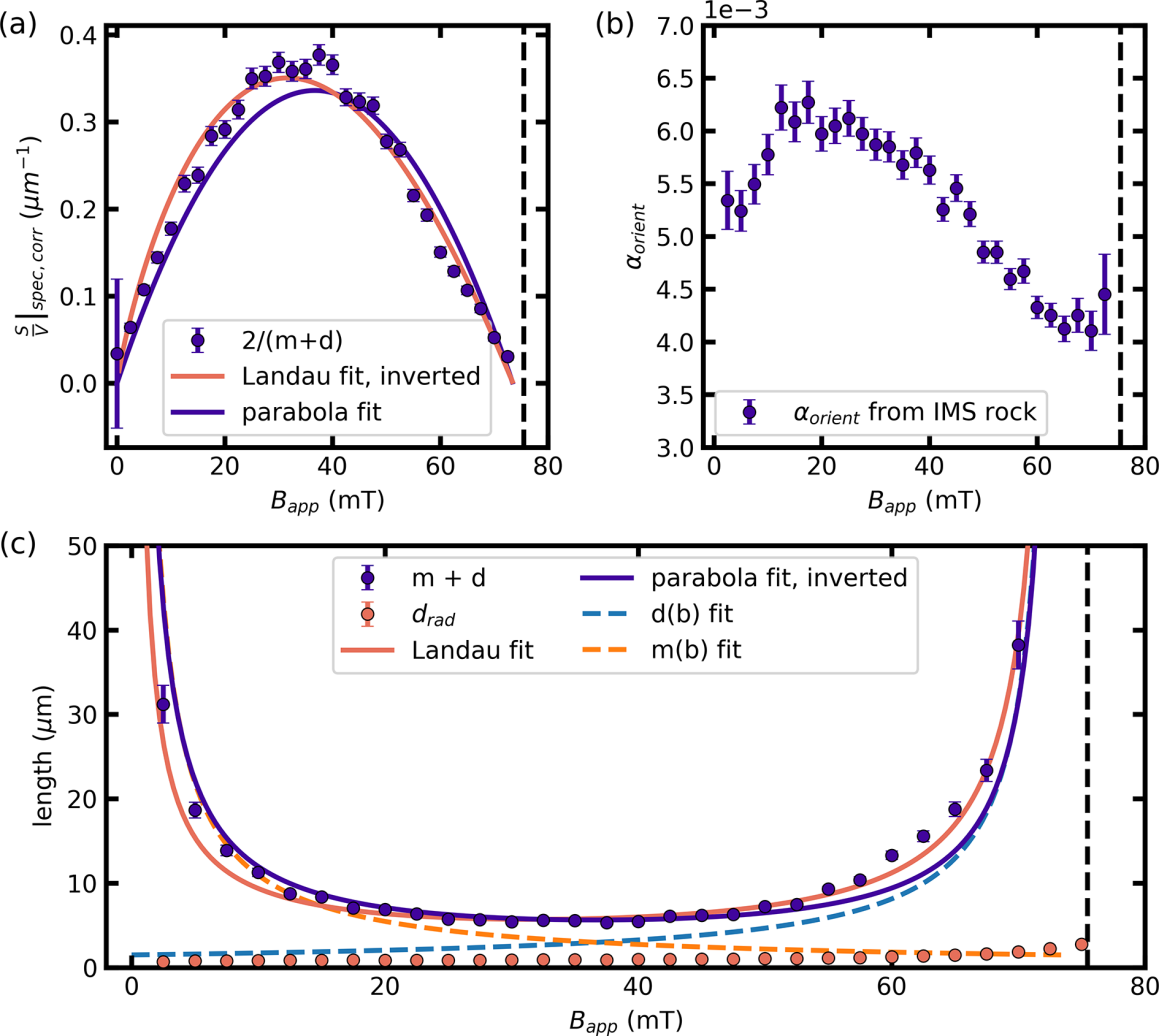
Corrected specific surface area 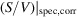 and repetition length *m* + *d*. (*a*) 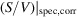 extracted from the low-*q* power-law scattering taking into account the aligned character of the IMS. (*b*) Correction factor α_orient_ obtained via equation (14). (*c*) Repetition length *m* + *d*, radial correlation length ξ_rad_ and *d*_rad_ extracted from ξ_rad_ according to equation (18). The solid lines in (*a*), (*c*) represent fits and their inverted fits to Landau’s model of domains (purple) and a parabola (orange) representing a simple model of the functional dependence of the sum of the size of mixed state domains *d* (blue dashed line) and Meissner state domains *m* (orange dashed line).

Fig. 8 ▸(*a*) shows 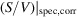 calculated from 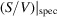 and α_orient_ according to equation (13). We only show data for fields *B*_app_ < B* as outside the IMS regime no low-*q* scattering is observed [see Figs. 5 ▸(*e*), 5 ▸(*f*)]. The correction factor α_orient_ is calculated according to equation (14) and shown in Fig. 8 ▸(*b*). For fields *B*_app_ = 0 and *B*_app_ > 75 mT the correction factor had an error larger than 15% and is not included in the following analysis. The correction factor is of the order of magnitude of ∼5 × 10^−3^ for the remaining field values. Turning to the description of 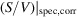, for both zero applied field and *B*_app_ = B*, 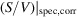 approaches zero. In between 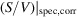 is close to symmetric around its maximum value of 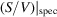
 = 0.38 µm^−1^ at *B*_app_ ≃ 0.5B*. The symmetric behavior can be fitted using a parabola, shown as the purple solid line.

Rearranging equation (16) allows us to extract the repetition length *m* + *d* shown in Fig. 8 ▸(*c*). *m* + *d* diverges at both ends of the IMS regime with extracted values close to 40 µm. We observe a nearly flat field dependence at intermediate field values in the range 20–50 mT, with a value of *m* + *d* = 5.5 µm at *B*_app_ ≃ 0.5B*.

## 5. Discussion

Having presented the results in the previous section, we will quickly summarize our main findings, further develop and compare our results with previous work and discuss some limitations of our analysis approach.

The internal field *B*_int_ shows a constant value below the transition field B*. This is a well known hallmark of the IMS and a result of the repulsive flux line interaction with an attractive tail leading to a constant inter flux line distance and hence a constant value of *B*_int_ inside the mixed state domains. The value of *B*_int_ agrees well with values obtained in previous studies on samples cut from the same single crystal (Brems *et al.*, 2022 ▸; Backs *et al.*, 2019 ▸).

The transition to the IMS can also be traced via the FLL correlation lengths in the three space directions: we observe a rapid decrease at the onset of the breaking up of the homogeneous FLL. ξ_tang_ and ξ_rad_ show very similar behavior and almost lie on top of each other. This indicates that the size of the mixed state domains in the plane orthogonal to the field is independent of the direction. In other words, the size of FLL domains is isotropic with respect to the orientation of the FLL, as can also be seen from the isotropic low-*q* scattering.

We have extracted the repetition length *m* + *d* over the entire field range of the IMS. The values of *m* + *d* have the same order of magnitude as values obtained via an analysis of the correlation peak of the domain structure from USANS measurements (Reimann *et al.*, 2017 ▸), indicating that the proposed modified Porod analysis yields physically meaningful results. Comparing the values of *m* + *d* with the radial correlation length ξ_rad_ leads to a similar conclusion: at *B*_app_ ≃ 0.5B*, the value is *m* + *d* = 5.5 µm. Based on the symmetric shape of *m* + *d* as a function of *B*_app_, a simple assumption is that *m* ≃ *d* = 0.5(*m* + *d*) ≃ 2.8 µm at *B*_app_ = 0.5B*. We find a value of ξ_rad_ = 0.8 µm, which is slightly smaller but on the same order of magnitude. We now further discuss our results with a focus on FLL correlation lengths, the domain sizes and *m* + *d* derived from the modified Porod analysis.

In the IMS, ξ_rad_ contains the contribution of the finite FLL perfection as well as the reduced size of the well ordered FLL due to the actual size of the mixed state domains. In the following, we present an attempt to remove the finite lattice perfection from ξ_rad_ to yield the actual size of the mixed state domains. Our observation of a finite plateau of ξ_rad_ for *B*_app_ > B* suggests that even in the conventional mixed state the extent of the well ordered FLL has an upper limit. Under the assumption that the contribution of the finite lattice perfection and the finite size of the FLL are independent of each other, we can calculate the size of mixed state domains projected in the horizontal direction *d*_rad_ according to 
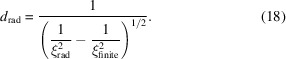
In equation (18) we assumed that the individual contributions of the finite size and perfection to the overall Bragg peak width are summed quadratically and that we can take the average value of the FLL perfection in the mixed state ξ_finite_ ≃ 2.2 µm as the value for the maximum achievable FLL perfection in the IMS.

The results are shown in Figs. 7 ▸(*b*) and 8 ▸(*c*) as orange dots. For low applied fields the field dependence of *d*_rad_ is qualitatively equivalent to that observed for ξ_rad_. For increasing fields approaching B*, *d*_rad_ is now rapidly increasing, as expected for a decreasing size of the Meissner state domains. As discussed in Section 3.2.2, the exact values of ξ_*i*_ and their derived quantities are subjective and depend on the exact definition of the correlation length. We defined the correlation length as ξ = 2π/FWHM = 2π/σ_*i*_*k* in analogy to the Scherrer equation used to quantify the sizes of crystallites in powder diffraction experiments (Scherrer, 1918 ▸). In addition to the subjectivity for the definition of ξ, an individual mixed state domain could contain more than one crystallite or contain voids. The assumption of the lattice perfection being independent of the mixed state domain size might be too simplistic and adds to the uncertainty of the extracted value of *d*_rad_. Therefore, the extracted size *d*_rad_ should be seen as an order of magnitude of the average size of the mixed state domains.

Furthermore, we stress that the obtained value of *d*_rad_ represents only the projected average length of mixed state domains in the direction parallel to **q**, as schematically depicted in the inset of Fig. 3 ▸.

The average size *d* of the mixed state domains over all directions is contained in the domain structure repetition length *m* + *d*, discussed in the following. We now turn to the discussion of the repetition length, domain sizes and other quantities extracted from the modified Porod analysis.

**Figure 9:**
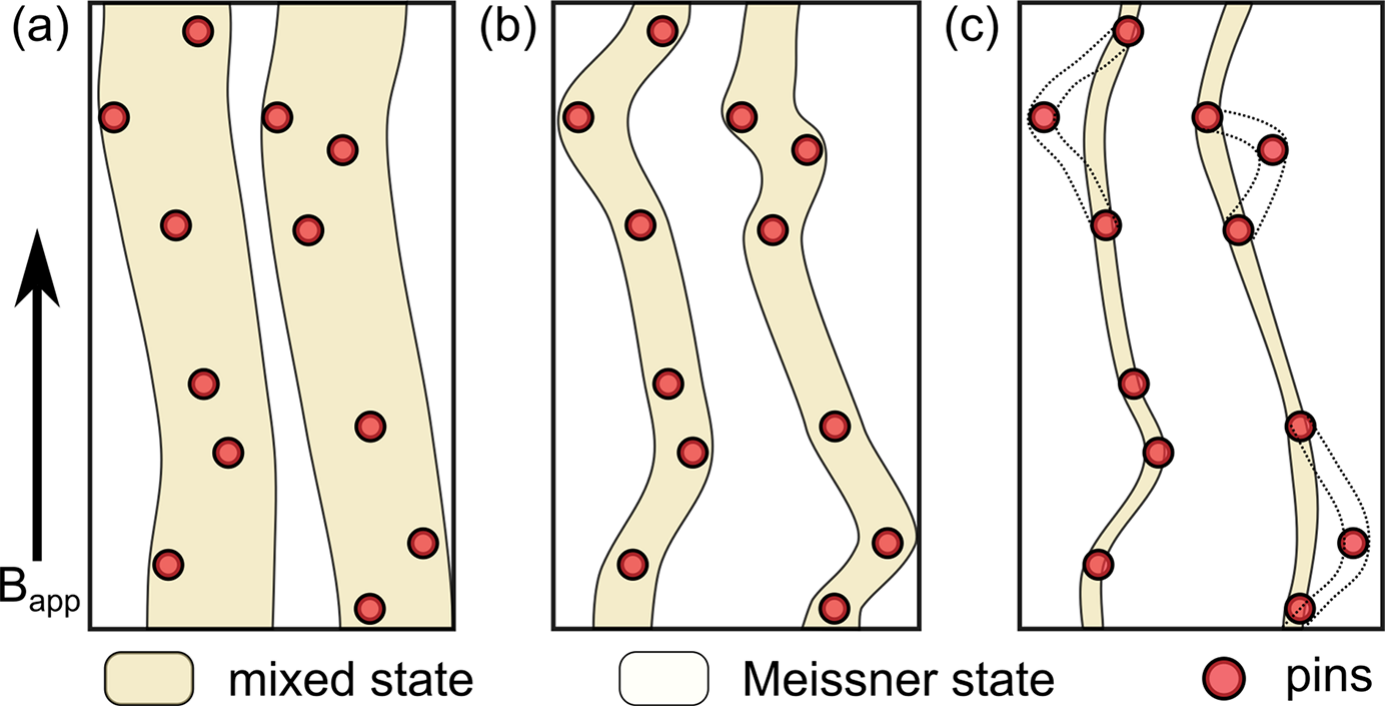
Schematic illustration of the field-dependent curvature of mixed state domains along the field direction which is directly related to σ_IMS_ used in the calculation of α_orient_. (*a*) For high values of *B*_app_ the domains are large and the influence of the pins on the straightness along the field direction is limited. (*b*) For intermediate field values the mixed state domains are increasingly bent due to the interaction with the pinning landscape. This leads to an increase in σ_IMS_ and α_orient_ with decreasing field. (*c*) For low fields approaching zero, the maximum curvature determined by the elastic moduli prohibits the accommodation of all pins, leading to an increased straightness and decreasing values of σ_IMS_ and α_orient_ as compared with intermediate field values. The schematic drawings are exaggerated and not to scale.

The repetition length *m* + *d* is calculated using the correction factor α_orient_. We observe a non-trivial field dependence of α_orient_, showing a slight peak for intermediate fields in the IMS [see Fig. 8 ▸(*b*)]. The correction factor is directly determined by the width of the IMS rocking scan σ_IMS_, which is linked to the straightness of the domain boundaries along the field direction. A possible explanation for this non-trivial behavior can be found when considering the influence of the pinning landscape on the straightness of the mixed state domains as a function of field. This is shown in Fig. 9 ▸, which schematically depicts the mixed state domain straightness in the field direction for three different fields. The interaction of the mixed state domain boundaries with the pinning landscape is governed by the elastic properties of the FLL. It can be quantified in analogy to crystal lattices by the elastic moduli of the FLL determined by the flux line interaction (Brandt, 1995 ▸). The elastic moduli set an upper limit for the curvature of the FLL along the field direction to accommodate a pin. In the IMS, where the inter flux line spacing is independent of the applied field, the elastic moduli can be considered close to constant. This results in a global maximum curvature of the flux lines over the entire field range of the IMS. The maximum curvature can be expressed as a length scale.

For a given pinning landscape, the curvature of the flux lines along the field direction is then solely determined by *d*, as schematically depicted in Fig. 9 ▸. For large *d* at high fields, the curvature is small and the domain boundaries are close to parallel to the field direction. For decreasing field the curvature of the domains increases as *d* decreases, translating to larger values of σ_IMS_ and α_orient_. Below a certain threshold value and consequently smaller values of *d*, the maximum curvature of the flux lines is reached. As a result, the value of σ_IMS_ for low fields is larger as compared with intermediate field values, explaining the non-trivial field dependence of α_orient_.

Recently (Reimann *et al.*, 2017 ▸), the repetition length of the IMS domain structure has been modeled using Landau’s theory (Landau, 1937 ▸) for domain structures in superconductors,
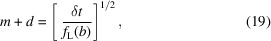
with the thickness of the superconducting sample *t*, reduced field *b*, superconducting wall-energy parameter δ and *f*_L_ a numerical function of which an explicit formulation, given by Dorsey & Goldstein (1998 ▸), was used.^3^ In contrast to type-I superconductors, where δ is a measure of the superconducting surface energy, here it describes a cleavage energy of the FLL due to the partially attractive flux line interaction (Reimann *et al.*, 2017 ▸).

For the IMS *b* has been adapted to 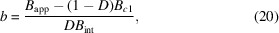
to take into account the demagnetization effects due to the demagnetization factor *D* (Reimann *et al.*, 2017 ▸). In our case, where the IMS covers the entire range of fields of 0 < *B*_app_ < B* and no Meissner state is observed, a definition of *b* according to equation (20) is not applicable as it fails to capture the whole range of the IMS, especially in the low applied field range. Therefore, we use a simplified version given by 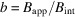. This is justified by the absence of a Meissner state following the FC measurement procedure due to macroscopic flux trapping. With the above adaptation of Landau’s theory, we can fit the repetition length *m* + *d* obtained from 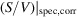 to obtain a value of δ = 7.4 ± 0.1 Å, with the error determined by the uncertainty of the fit. The fit is shown as the solid orange line in Fig. 8 ▸(*c*). The value obtained by Reimann *et al.* (2017 ▸) is δ = 13 ± 2 Å using a sample of different shape and purity. The sample thickness is taken into account in equation (19), but it is hard to quantify the influence of the sample purity. Nevertheless, the comparison with our results shows that our analysis approach yields the same order of magnitude.

The repetition length is given as the sum of Meissner length *m* and the mixed state length *d* as *m* + *d*. On the basis of the symmetric shape of *m* + *d* around *b* = 0.5, we develop a purely phenomenological model taking into account the asymptotic *b* dependence of the Meissner length *m* and the mixed state length *d*:
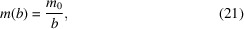

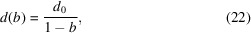
where *m*_0_ and *d*_0_ are constant factors.

As we are extracting only the length *m* + *d*, we need a model function to connect our *ansatz* from equations (21) and (22) to 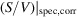. The symmetric shape of 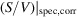 can be fitted with a parabola of the form 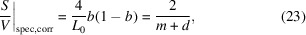
where *L*_0_ is a constant scaling factor with the dimension of a length following from equation (16).

If we invert equation (23) and insert our *ansatz* from equations (21) and (22), we can deduce explicit forms for *m*(*b*) and *d*(*b*): 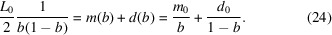
For the last equation to hold we see that *m*_0_ = *d*_0_ and 2*m*_0_ = *L*_0_.

Considering the symmetric shape of 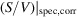 around *b* = *B*_app_/*B*_int_ = 0.5 yields an intuitive explanation of the requirement *m*_0_ = *d*_0_: for a volume filling fraction of 50% the mixed state length *d*(*b*) is equal to the Meissner state length *m*(*b*).

We thus get 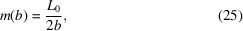

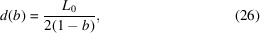
with *L*_0_ the only free parameter. We fitted equation (23) to 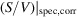 to extract *L*_0_. The result is shown as the solid purple line in Fig. 8 ▸(*a*). The fitted value with the error determined by the fit is *L*_0_ = (2.9 ± 0.1) µm. After extracting *L*_0_ we can use equations (25) and (26) to plot the functional dependence of *m* and *d*. The results are shown as dashed lines in Fig. 8 ▸(*c*).

We see that there is an overall qualitative agreement of *d*(*b*) obtained from our modified Porod analysis and *d*_rad_ from the Bragg peak width, both showing rapidly increasing behavior when approaching B* and a constant value for low fields. A quantitative agreement can not be seen, partially because the correlation length is only an approximation of the mixed state domain size, as discussed above.

We conclude this section by discussing some limitations in our analysis approach. We extract the FLL domain size from both the Bragg peak widths and the low-*q* Porod scattering. Our analysis of the Porod scattering is based on a simple modification of the Porod law to take into account the alignment of the scattering surfaces orthogonal to the neutron beam by introducing a correction factor α_orient_. Via simple geometry, the repetition length *m* + *d* is then obtained from the corrected specific surface area from the slope of the radially averaged scattering data in absolute scattering intensities. Slight systematic errors in the data reduction introduced by the choice of the region of interest in the calculation of the normalization and calibration constants (transmission, direct beam flux *etc.*) can influence the fitted slope of the intensity curves. Other sources of possible errors are the choice of the correction factor α_orient_ and the simple assumption of virtual rearrangement in the calculation of *m* + *d* from 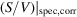. Finally, the model presented in equations (21) and (22) might be oversimplified to capture the actual field dependence of the mixed state domain size, as can be seen by deviations between the experimental data of 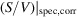 and the fit shown in Fig. 8 ▸. We extracted correlation lengths from the Bragg peak widths to estimate the contribution of the finite size broadening. In our analysis, we assumed that the intrinsic FLL perfection is independent of the mixed state domain size. Furthermore, *d*_rad_ only represents the projection of *d* along the direction of **q**.

Despite these sources of uncertainty, we are confident that the overall order of magnitude of the extracted length scales from both the Porod analysis and the Bragg peak widths is correct. This is shown by the qualitative agreement of our two analysis approaches, the overall agreement with previous work (Reimann *et al.*, 2017 ▸) and Landau’s theory of domains in superconductors (Landau, 1937 ▸).

## 6. Conclusion

We have presented a novel approach based on a modification of the well known Porod law to extend the accessible length scales to up to 40 µm in a classical SANS experiment.

Our analysis approach is based on the alignment of the two-phase domain structure along the beam direction. The degree of alignment is directly measured via a rocking scan and used to extract the corrected specific surface area, allowing the calculation of the mean repetition length *m* + *d*. In principle, the proposed analysis approach is applicable to any degree of alignment along the beam direction, with the case of isotropically distributed surfaces resulting in the conventional Porod law. We note that even in the case of the conventional Porod law the specific surface area can still be connected to the mean interparticle distance, but only if the particle dimensions are known. In the special case of an aligned structure where the domain surfaces continuously extend through the sample, we have shown that the specific surface area is linked directly to a mean intersurface distance.

We apply our method to the two-phase domain structure found in the IMS of the superconductor niobium, where the domain boundaries are aligned along the beam direction. We extract the repetition length of the domain structure *m* + *d* over the entire field range.

Our results are consistent with previous attempts to derive this length scale from USANS (Reimann *et al.*, 2017 ▸), Landau’s theory of domains in superconductors (Landau, 1937 ▸) and the well established method of extracting correlation lengths from Bragg peak widths (Cubitt *et al.*, 1992 ▸; Cubitt, 1994 ▸; Grigoriev *et al.*, 2010 ▸) that can be connected to the finite size of the FLL domains. The overall agreement showcases the power of our analysis approach in extending the accessible length scales from 1 µm by almost two orders of magnitude to up to 40 µm in a conventional SANS setup without compromising the experiment by long counting times. Our study can act as a proof of concept for use in other systems showing alignment along the beam direction with unbroken surfaces extending through the length of the sample.

## Footnotes

1 Taking into consideration the finite pixel size of detectors, the leading term in the observed scattering intensity is still proportional to *q*^−4^ even for particles with non-convex surfaces, possibly also including edges, vertices and planar facets (Schneider *et al.*, 2002 ▸).

2 For comparison, the nuclear scattering length density of D_2_O can be calculated to be ρ_D2O_ = 6.4 × 10^−6^ Å^−2^ using https://www.ncnr.nist.gov/resources/activation/ with a mass density of  = 1.11 g cm^−3^ for D_2_O.

3 We note that there is an error in equation (7) of Reimann *et al.* (2017 ▸). We present the correct version, which is in agreement with, *e.g.*, equation (3.23) of Dorsey & Goldstein (1998 ▸).
